# Evaluating AI-based mosquito monitoring technologies: a field study in Zhejiang Province, 2025

**DOI:** 10.3389/fvets.2026.1831713

**Published:** 2026-06-04

**Authors:** Jinna Wang, Kexin Wang, Mingyu Luo, Tianqi Li, Qinmei Liu, Zhou Guan, Zhenyu Gong, Jimin Sun

**Affiliations:** 1 Zhejiang Provincial Center for Disease Control and Prevention, Hangzhou, China; 2 Yiwu Center for Disease Control and Prevention, Yiwu, China

**Keywords:** AI, intelligent, mosquito, mosquito-borne disease, surveillance

## Abstract

**Objectives:**

This study aimed to assess the effectiveness of intelligent mosquito surveillance devices implemented in Zhejiang Province and to compare these metrics with those obtained from traditional surveillance methods.

**Methods:**

Intelligent surveillance devices, light traps, and BG-traps were operated concurrently at the same locations. Statistical comparisons were conducted using one-way ANOVA with Dunnett's T3 tests, while the Dynamic Time Warping algorithm was employed to evaluate similarities in density trends.

**Results:**

A total of 278 trap-nights using light traps and 278 trap-hours of BG-traps captured 11,349 and 1,524 mosquitoes, respectively, yielding mean densities of 14.56 mosquitoes/(trap·night) and 6.34 mosquitoes/(trap-hour). Additionally, 991 and 646 trap-days of Chengwen Jingling traps and CAMMADE traps recorded 12,003 and 1,180 mosquitoes, respectively, based on backend data, with mean densities of 5.62 mosquitoes/(trap-day) and 1.83 mosquitoes/(trap-day). The light trap produced significantly higher mosquito densities than all other methods (*P* < 0.05), while the BG-trap exhibited comparable densities to Chengwen Jingling traps (*P* > 0.05) but significantly outperformed the CAMMADE trap (*P* < 0.05). During the same time window on the same day, the trapping efficacy of the BG-trap was significantly higher than that of the Chengwen Jingling Trap (*P* < 0.05), while no significant difference was observed between the light trap and the Chengwen Jingling Trap (*P* > 0.05). Temporal pattern analysis further revealed consistent trends among the Chengwen Jingling, light trap, and BG-trap methods. The Chengwen Jingling trap exhibited relative error rates (RE) of 25.67% for density estimation, 64.29% for *Culex* genus identification, and 179.20% for *Aedes* genus identification. The CAMMADE trap demonstrated RE of 105.34% for density estimation, along with 15.99% for *Culex* and 34.69% for *Aedes* at the genus level. For female mosquito identification, Chengwen Jingling achieved RE of 21.54% for *Culex* and 76.72% for *Aedes*, while CAMMADE showed rates of 102.99% for *Culex* and 153.85% for *Aedes*.

**Conclusions:**

AI-based mosquito monitoring technologies exhibit significant potential for advancing vector surveillance; however, current technical limitations in trapping efficacy and algorithm accuracy necessitate a hybrid approach integrating AI with conventional methods in the near term, rather than a full replacement.

## 1 Introduction

Mosquitoes are vectors for numerous diseases, including dengue fever (DF), chikungunya fever (CHIKF), malaria, Zika virus disease, and Japanese encephalitis (JE) (1, 2), thereby posing a significant threat to human health. The World Health Organization ranks mosquitoes as the deadliest animals, with the annual number of affected individuals surpassing the combined total of all other animals on the list (3). DF is an acute infectious disease primarily caused by the dengue virus and transmitted by *Aedes albopictus* (*Ae. albopictus*) and *Aedes aegypti* (*Ae. Aegypti*) (4). With the global expansion of *Aedes* mosquitoes driven by climate change and globalization, DF importation poses an emerging threat to non-endemic regions with susceptible populations (5). In 2024, global DF cases surged to 14.1 million, doubling the historic 7 million cases reported in 2023 and representing a 12-fold increase compared to 2014, with 9,508 associated deaths recorded (6). CHIKF caused by the chikungunya virus, is an acute infectious disease transmitted primarily by *Ae. albopictus* and *Ae. aegypti* (7). In 2025, the global epidemic of CHIKF was severe, with an outbreak reported in Guangdong Province, China, leading to a significant disease burden (8). In addition to *Aedes* mosquitoes, *Culex* species also serve as important disease vectors; for instance, *Culex tritaeniorhynchus* (*Cx. tritaeniorhynchus*) is a known vector of JE (9). Evidence indicates that JE remains a severe disease affecting both adults and children, frequently resulting in substantial neurological sequelae despite current clinical management practices (10). Mosquito-borne infectious diseases represent a considerable threat to public health, contributing to a disproportionately high global disease burden (11). Therefore, the prevention and control of these diseases are essential public health priorities.

Currently, effective therapeutic interventions and prophylactic vaccines are unavailable for most mosquito-borne diseases, making vector control the cornerstone of prevention and control strategies (12). The establishment of a sensitive, closed-loop surveillance system is an urgent priority to facilitate real-time tracking of mosquito density, thereby effectively mitigating vector populations and the risk of disease transmission. At present, mosquito density monitoring in China primarily relies on the national standard “Surveillance Methods for Vector Density-Mosquitoes” (GB/T 23797-2020) (13). Adult mosquito density monitoring methods primarily encompass the light trap method, the Biogents (BG) trap method, gravid female mosquito trap method, human landing catch method, and double net trap method. These monitoring techniques continue to depend on traditional, labor-intensive approaches and standalone equipment, which present several significant drawbacks. Coordination among multiple personnel is necessary for the deployment and retrieval of instruments. Additionally, captured specimens must be transported to laboratories for manual sorting, counting, and identification under dissecting microscopes, placing a burden on frontline staff. Furthermore, the functional limitations of current surveillance equipment are evident across multiple dimensions. Most monitoring stations predominantly utilize light traps, a method primarily effective for *Culex* species; however, these traps exhibit limited attraction efficacy for diurnal species such as *Ae. albopictus*, as their phototactic design principally targets nocturnal activity patterns (14). Conversely, the BG-Trap method, while demonstrating superior performance for *Ae. albopictus* through its optimized CO_2_ and kairomone-based attraction mechanisms, performs poorly for other species—particularly nocturnal *Culex* mosquitoes that rely on phototactic cues rather than host-seeking olfactory signals (15, 16). This methodological selectivity necessitates the deployment of multiple trap types to achieve comprehensive vector surveillance coverage, substantially increasing operational complexity and resource requirements. Furthermore, the workflow encompassing field deployment, specimen identification, data entry, and hierarchical reporting typically requires several days. This delay impedes the timely detection of high-density infestations and obstructs the integration of these findings with dengue transmission risk models and precision control systems.

Recent advancements in the deep integration of artificial intelligence, the Internet of Things, big data, and robotics have created new opportunities for mosquito surveillance and disease prevention (17). The development and deployment of Artificial Intelligence (AI) -powered monitoring devices are becoming increasingly prevalent (18). Brazilian researchers developed an intelligent trap that integrates Internet of Things (IoT) technology, high-resolution cameras, and You Only Look Once version (YOLOv7) algorithms for the real-time monitoring and control of *Ae. aegypti*, thereby significantly enhancing the accuracy of dengue vector surveillance (19). Italian researchers assessed the performance of the Vector Tracking (VECTRACK) system in terms of smart trapping efficiency, species identification, sex recognition, and capture rates of *Ae. albopictus* (20). Sanavria A, et al. employed the Intelligent Dengue Monitoring and Intelligent Virus Monitoring systems to detect the presence of female *Ae. aegypti* in the municipality of Seropédica, Rio de Janeiro State (21). Uelmen JA Jr, et al. developed the Global Mosquito Observations Dashboard (GMOD), a user-friendly web interface that utilizes citizen science to monitor invasive and vector mosquito species (22). Sauer FG et al. reported that their most effective convolutional neural network (CNN) model achieved 99% precision in differentiating *Aedes* from non-*Aedes* mosquitoes and 91% mean precision in classifying *Aedes* species using red-green-blue (RGB) imagery (23). Additionally, another study revealed that VectorCam, an imaging hardware and mobile application system developed for mosquito species identification, served as a practical tool for simplifying field-based mosquito specimen identification among village health teams in rural Uganda (24). A review has underscored the advancements in mosquito behavioral monitoring tools over the past decade, facilitated by state-of-the-art video surveillance and artificial intelligence. These advancements include monitoring flight behavior for three-dimensional pattern analysis, conducting high-throughput egg counting for fecundity studies, and observing feeding behaviors for detailed examinations (25). This transition from manual deployment and counting to AI recognition paired with IoT real-time sensing signifies a future trend in the control of mosquito-borne diseases such as DF and CHIKF.

Zhejiang Province, situated in China's southeastern coastal region, possesses climate conditions that are highly conducive to the growth, development, and reproduction of mosquitoes. Periodic outbreaks of mosquito-borne infectious diseases, particularly DF and CHIKF, pose significant threats to public health and impose a considerable burden on the local health care system (26, 27). In response to the limitations of existing mosquito surveillance methods, the Zhejiang Provincial Center for Disease Control and Prevention (CDC) initiated a pilot field project in 2025 focused on intelligent mosquito surveillance. This project aims to leverage the AI, IoT, and big-data technologies to establish a fundamentally new paradigm for vector monitoring and to evaluate its effectiveness in comparison to traditional methods.

## 2 Materials and methods

### 2.1 Study design

**Figure 1:**
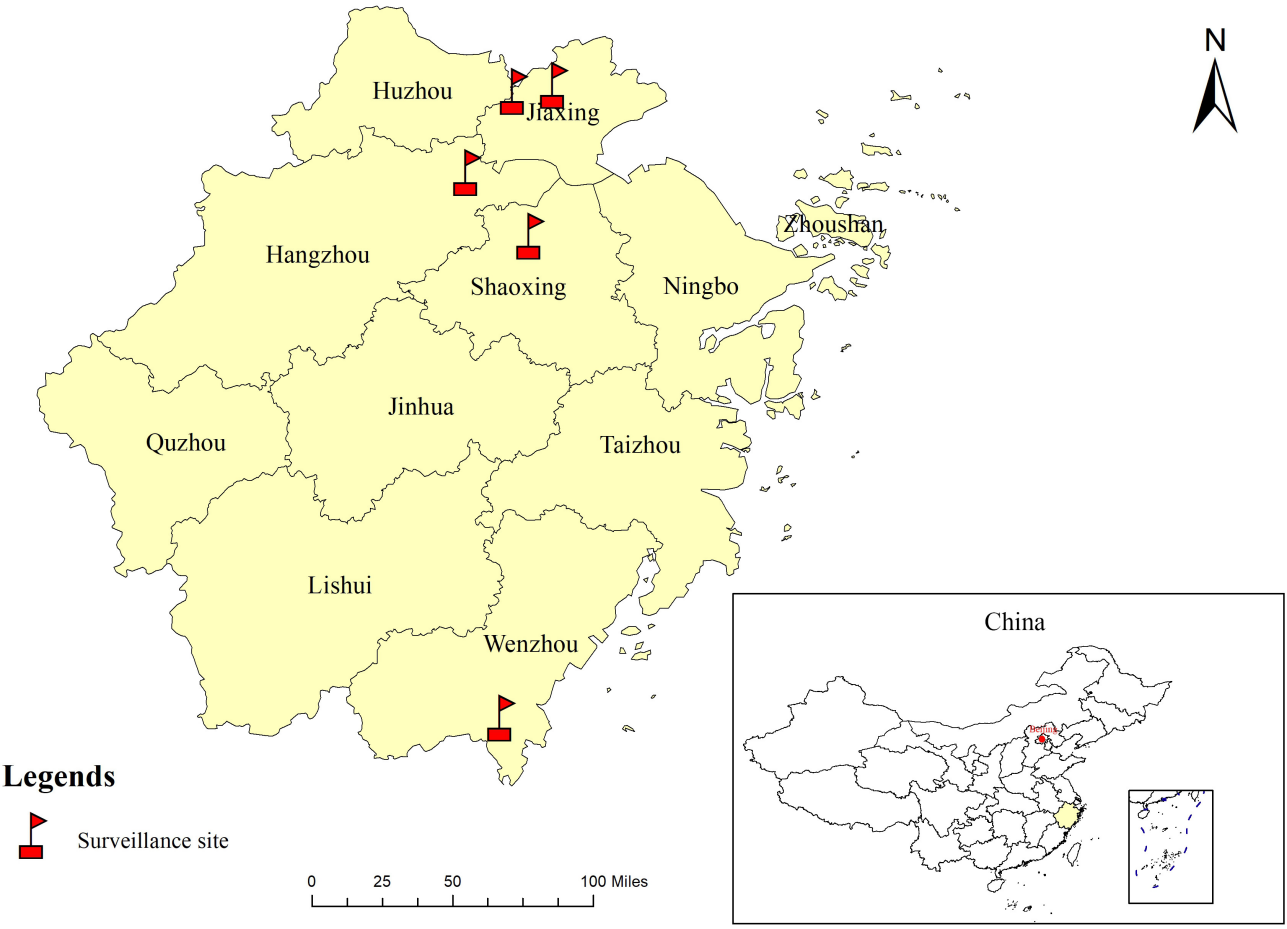
Locations of the five surveillance sites in Zhejiang Province, 2025.

**Figure 2:**
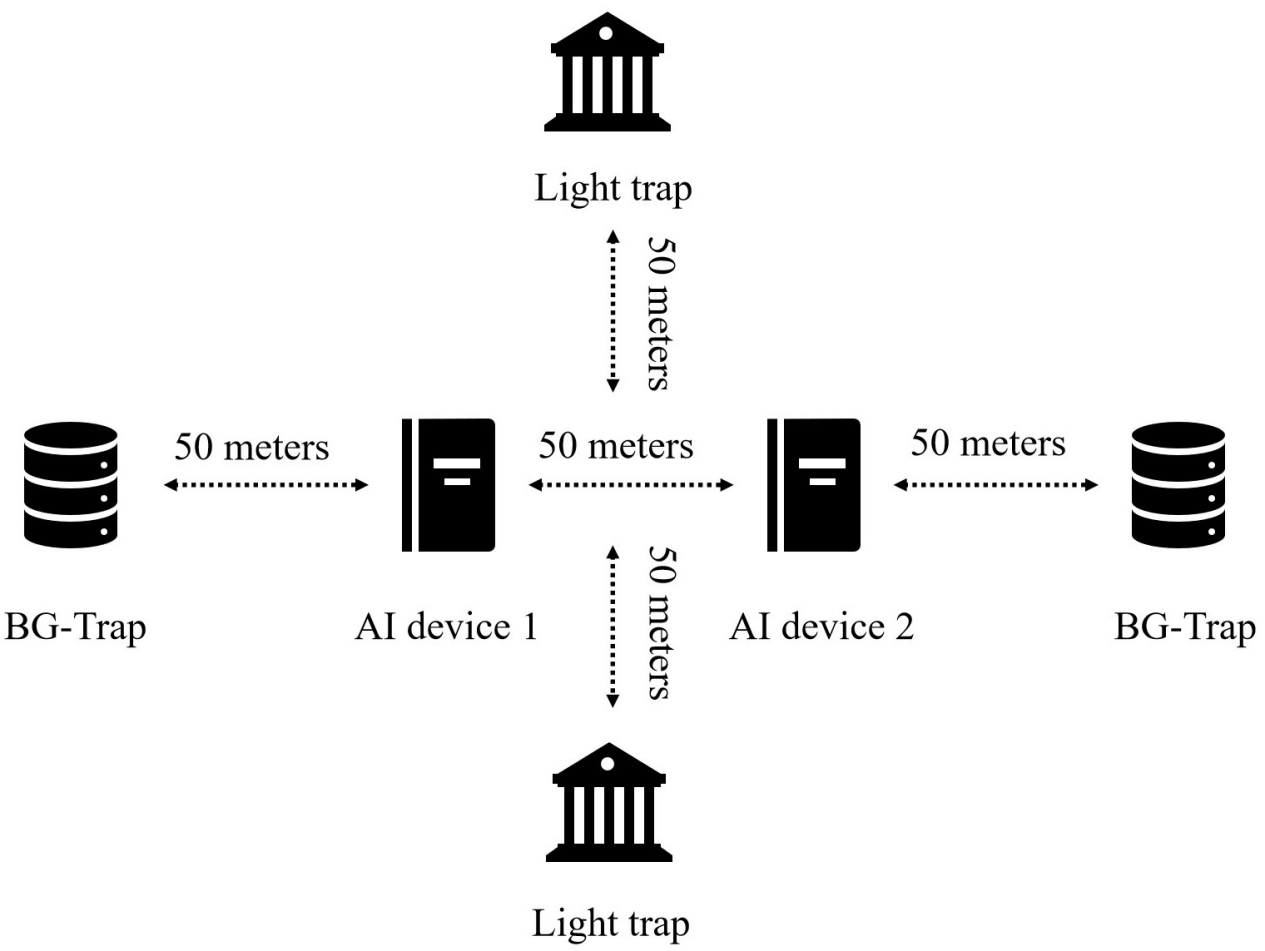
Schematic diagram of trap locations at each surveillance site.

Considering epidemiological profiles, environmental suitability, and technical compatibility, we identified areas with historically high mosquito densities or frequent outbreaks of mosquito-borne diseases as surveillance sites. Ultimately, five locations were selected: Shangcheng District (Hangzhou city), Yuecheng District (Shaoxing city), Cangnan County (Wenzhou city), Xiuzhou District and Tongxiang City (Jiaxing city) (Figure 1). At each monitoring site, a single rural residential area or community was selected for the pilot study. Eligible villages or communities were required to comprise a minimum of 500 households with a permanent resident population of ≥1,000. Mosquito monitoring sites were located in sheltered and shaded outdoor areas away from interfering light sources, such as green belts and other locations preferred by mosquitoes for resting. At each of these sentinel sites, intelligent mosquito-monitoring devices were deployed to continuously assess mosquito density. Simultaneously, conventional mosquito-density surveillance methods, including the light trap method and BG-trap method were implemented weekly at the same locations. To ensure comparability across surveillance methods, the conventional mosquito-density surveillance methods were deployed at each monitoring site on the same day of each week at identical locations, establishing a standardized comparative benchmark. To prevent mutual interference, devices were positioned 50 meters apart (Figure 2). The monitoring period began in May 2025 and concluded at the end of November 2025.

### 2.2 The intelligent mosquito surveillance method

Based on preliminary research and pre-experimental findings concerning intelligent mosquito monitoring products, two models of intelligent mosquito surveillance devices were selected: the Chengwen Jingling Intelligent Adult Mosquito Surveillance Trap (Chengwen Jingling Trap, MA-E3, PASSONBIO, China) and the CAMMADE Intelligent Adult Mosquito Surveillance Trap (CAMMADE Trap, KMT001, Zhejiang Senhu Xi Health Technology Co., Ltd., China). The Chengwen Jingling Trap (40 × 40 × 116 cm, 13 kg) employs a multi-modal attraction system combining synthetic human kairomones with Ultraviolet (UV) light (360–420 nm). Captured specimens are drawn via suction fan and photographed (≥12-megapixel camera) for backend AI-based identification and sexing. The unit operates on Alternating Current (AC) 220V or Direct Current (DC) 12V battery (≥72 h continuous monitoring), with integrated environmental sensors for temperature, humidity, and wind speed. Real-time data transmission is enabled via a 5G IoT module with GPS/BeiDou positioning, supporting remote operation and monitoring through desktop and mobile platforms. A CNN-based AI system was constructed with a three-tier architecture (backbone-neck-head), where 2 × 2 convolutional kernels and partial self-attention (PSA) modules were incorporated in the head to enhance robustness. The model was trained with increased epochs and optimized loss functions. Deployed within an edge-cloud framework, the system performs real-time mosquito genus and sex identification at the edge, with structured data subsequently uploaded to the cloud for automatic calculation. The CAMMADE Trap integrates UV light (365 nm, 10 W), CO_2_ release (2 L high-pressure cylinder), and a 2500 V high-voltage grid for mosquito capture. A 5-megapixel camera enables automated imaging and identification based on deep learning algorithms, with data transmission via 4G IoT module and GPS/BeiDou positioning. The unit operates on AC 220 V or a 250 Wh battery (48-h capacity), housed in a rainproof (IPX4) metal enclosure (40 × 40 × 108 cm) with lockable casters for field deployment. The intelligent mosquito surveillance system adopted a four-layer architecture (perception, network, platform, and application), integrating image sensors, acoustic sensors, environmental sensors, and trapping devices. Edge computing performed real-time AI inference with <2-s latency, while the cloud handled storage, model training, and analytics. The system achieved target detection mean Average Precision (mAP) ≥90% (Intersection over Union (IoU) = 0.5) on independent test datasets. Automated functions included density index calculation, threshold-based alerting, environmental risk prediction, and intelligent trap control linked to monitoring results. Validation encompassed performance benchmarking, functional verification, continuous reliability testing (Mean Time Between Failures ≥10,000 h), and environmental adaptability assessments under IP65-rated conditions.

At each monitoring site, one unit per devices was deployed (Figure 2). The intelligent mosquito surveillance devices were placed on the ground in shaded, wind-sheltered locations, away from interfering light sources, and operated continuously (24 h d^−1^), automatically identifying and counting mosquitoes while generating density readings every hour. Staff from the local CDC collected the mosquitoes captured by the intelligent monitoring devices on a weekly basis. The specimens were subsequently transported to the laboratory for identification and counting through manual verification under a stereomicroscope, based on morphological characteristics using the dichotomous key from The Identification Pictures of Common Medical Vectors in Chinese Ports (28). The mosquito density was calculated as the number of female mosquitoes captured per trap per day.

### 2.3 The light trap method

Two light traps (Gongfu Xiaoshuai Photocatalytic Mosquito Trap, Wuhan Jixing Environmental Technology Co., Ltd., China) were deployed weekly at each monitoring site to assess adult mosquito density. Locations far from interfering light sources and sheltered from the wind were chosen as the surveillance sites. The light traps were positioned 1.5 m above the ground, activated 1 h before sunset, and remained operational until 1 h after sunrise the following day. Captured mosquitoes were identified and counted in the laboratory. The mosquito density was calculated as the number of female mosquitoes captured per light trap per night.

### 2.4 The BG-trap method

Two BG-traps (BG-Mosquitaire CO_2_, Biogents AG, Germany) were deployed weekly at each monitoring site. Each trap was baited with a steel cylinder that emitted CO_2_ at a rate of 500 g/24 h. The trap was positioned on the ground, with the BG-Lure (Biogents AG, Germany) placed in the designated pocket within the trap, while the steel cylinder was set beside it. BG-traps were operated during the peak activity period of *Ae. albopictus* (15:00–17:00), with each monitoring session lasting 30 min, ensuring that traps were spaced more than 50 meters apart. All captured mosquitoes were collected and identified morphologically to species. The mosquito density was calculated as the number of female mosquitoes caught per trap per hour.

### 2.5 Statistical analysis

The comparative analysis was conducted utilizing one-way ANOVA. Pairwise comparisons were executed using Dunnett's T3 test. The Dynamic Time Warping (DTW) algorithm facilitated the comparison of trends in mosquito density variation. All descriptive statistics and plots were generated using R version 4.0.2 (The R Foundation for Statistical Computing) and WPS Office 2023. Statistical significance was established at *P* < 0.05.

## 3 Results

### 3.1 Mosquito surveillance results

A total of 11,349 mosquitoes were captured using the light trap method, of which 4,047 (35.66%) were female. *Culex* species accounted for 97.14% of the catch, followed by *Aedes* species (2.69%) and *Anopheles* species (0.08%). The BG-Trap method captured 1,524 mosquitoes, with 881 females (57.81%). *Aedes* species represented 75.19% and *Culex* 24.08%. According to backend data from Chengwen Jingling Traps, 12,003 adult mosquitoes were recorded, including 5,573 females (46.43%). *Culex* accounted for 86.94% and *Aedes* for 12.76%. The CAMMADE traps recorded 1,180 mosquitoes in the backend system. These traps lack sex identification capability, so all captured mosquitoes were presumed to be female by default. *Culex* species accounted for 94.41% and *Aedes* for 5.59% (see Table 1 for details).

**Table 1:** Mosquito density surveillance results in Zhejiang Province, 2025.

Methods	Trap time	No. of mosquitoes	No. of female mosquitoes	Mosquito Density	*Culex* species		*Aedes* species		*Anopheles* species		Others	
					female	male	female	male	female	male	female	male
Light trap method (mosquitoes/(trap·night))	278	11,349	4,047	14.56	3,863	7,161	171	134	6	3	7	4
BG-Trap method (mosquitoes/(trap·hour))	278	1,524	881	6.34	240	127	639	516	0	0	2	0
Chengwen Jingling Trap (mosquitoes/(trap·day))	991	12,003	5,573	5.62	4,957	5,478	616	915	0	37	0	0
CAMMADE Trap (mosquitoes/(trap·day))	646	1,180	1,180^*^	1.83	1,114	0	66	0	0	0	0	0
Total	—	26,056	11,681	—	10,174	12,766	1,492	1,565	6	40	9	4

^*^CAMMADE trap: all captured mosquitoes are assumed to be female for density calculations, which may result in overestimation.

### 3.2 Comparison of mosquito densities among different surveillance methods

The average weekly mosquito density for each surveillance method was calculated using a working week (Monday–Sunday) as the unit, based on the current mosquito density unit. Comparative analysis among different methods was performed using one-way ANOVA after normality testing confirmed that the data met the distributional assumptions. The results are presented in Table 2. Significant differences in average weekly mosquito density were observed among the monitoring methods (*P* < 0.05).

**Table 2:** Comparison of mean weekly mosquito densities across different surveillance methods in Zhejiang Province in 2025.

Methods	*n*	M	SD	SE	95%CI		*F*	*P*
					LL	UL		
Light trap	30	15.13	10.82	1.98	11.09	19	24.306	<0.001
BG-trap	30	6.21	3.14	0.57	5.03	7.38		
Chengwen Jingling Trap	30	6.16	5.39	0.98	4.15	8.17		
CAMMADE Trap	30	1.59	1.56	0.29	1.01	2.18		
Total	120	7.27	7.93	0.72	5.84	8.71	—	—

M, mean; SD, standard deviation; SE, standard error; CI, confidence interval; lower limit, LL, upper limit=UL.

Pairwise comparisons of average weekly mosquito density among surveillance methods were conducted using Dunnett's T3 test due to unequal variances, with results presented in Table 3. Based on the current mosquito density units for each method, the light trap method produced significantly higher mosquito densities than all other surveillance methods (*P* < 0.05). Mean comparisons revealed that the light trap method's mosquito density was 2.44 times that of the BG-trap method, 2.46 times that of Chengwen Jingling trap method, and 9.52 times that of CAMMADE trap. The BG-trap method showed no statistically significant differences from the Chengwen Jingling trap method, but was significantly higher than CAMMADE trap method (*P* < 0.05). However, it should be noted that this comparison merely compared the differences in density values across different monitoring methods, even though the monitoring approaches, durations, and density units of each method were different.

**Table 3:** Results of pairwise comparisons of mean weekly mosquito densities across surveillance methods in Zhejiang Province.

Methods(I)	Methods(J)	M(I–J)	SE	*P*	95%CI	
					LL	UL
Light trap	BG-Trap	8.92^*^	2.06	0.001	3.19	14.65
	Chengwen Jingling Trap	8.97^*^	2.21	0.001	2.89	15.04
	CAMMADE Trap	13.53^*^	2.00	<0.001	7.94	19.13
BG-trap	Chengwen Jingling Trap	0.05	1.14	1.000	−3.08	3.17
	CAMMADE Trap	4.61^*^	0.64	<0.001	2.85	6.38
Chengwen Jingling trap	CAMMADE Trap	4.57^*^	1.02	0.001	1.72	7.42

^*^The significance level for mean differences was set at 0.05.

### 3.3 Standardized comparison of different surveillance methods

Comparison of mosquito trapping efficacy was done across surveillance methods. Since the mosquito density units differed among surveillance methods, densities were standardized to mosquitoes per trap per hour to enable comparison of trapping efficacy, with results presented in Table 4. Significant differences in trapping efficacy were observed among methods (*P* < 0.05). Pairwise comparisons were conducted using Dunnett's T3 test, with results shown in Table 5. The significant differences were detected among all method pairs (*P* < 0.05). Mean comparisons revealed that the BG-trap achieved the highest trapping efficacy, followed by the light trap method, and the Intelligent Mosquito Surveillance Trap has the lowest mosquito-capturing efficiency, with Chengwen Jingling trap demonstrating higher efficacy than CAMMADE Trap. The trapping efficacy of the BG-trap method was 5.75 times that of the light trap method, 23.88 times that of Chengwen Jingling trap, and 88.71 times that of the CAMMADE trap.

**Table 4:** Comparison of mosquito trapping efficacy among surveillance methods in Zhejiang Province in 2025.

Methods	*n*	M	SD	SE	95%CI		*F*	*P*
					LL	UL		
Light trap	30	1.08	0.77	0.14	0.79	1.37	95.968	<0.001
BG-trap	30	6.21	3.14	0.57	5.03	7.38		
Chengwen Jingling trap	30	0.26	0.22	0.04	0.17	0.34		
CAMMADE trap	30	0.07	0.07	0.01	0.04	0.09		
Total	120	1.90	2.99	0.27	1.36	2.44		

M, mean; SD, standard deviation; SE, standard error; CI, confidence interval; LL, lower limit; UL, upper limit.

**Table 5:** Pairwise comparison of mosquito trapping efficacy among surveillance methods in Zhejiang Province in 2025.

Methods(I)	Methods(J)	M(I-J)	SE	*P*	95%CI	
					LL	UL
Light trap	BG-trap	−5.13^*^	0.59	<0.001	−6.78	−3.48
	Chengwen Jingling trap	0.82^*^	0.15	<0.001	0.41	1.23
	CAMMADE trap	1.01^*^	0.14	<0.001	0.62	1.41
BG-Trap	Chengwen Jingling trap	5.95^*^	0.58	<0.001	4.33	7.57
	CAMMADE trap	6.14^*^	0.57	<0.001	4.53	7.75
Chengwen Jingling trap	CAMMADE trap	0.19^*^	0.04	0.001	0.07	0.31

^*^The significance level for mean differences was set at 0.05.

The monitoring periods of the intelligent mosquito surveillance method were further standardized by selecting data from the same time window on the same day for the intelligent mosquito trap, light trap, and BG-Trap. Because data from the CAMMADE trap could not be retrieved independently, only data from the Chengwen Jingling trap were analyzed. All calculation units were standardized as mosquitoes per trap per hour. The comparative results were presented in Tables 6, 7. The results showed that there was no statistically significant difference between the light trap and the Chengwen Jingling trap during the same time period (*P* > 0.05). However, the mosquito trapping efficacy of the BG-Trap was significantly higher than that of the Chengwen Jingling trap during the same time period (*P* < 0.05).

**Table 6:** Comparison of mosquito trapping efficacy among surveillance methods under concurrent operation periods.

Methods	n	M	SD	SE	95%CI		*F*	*P*
					LL	UL		
Light trap	30	1.08	0.77	0.14	0.79	1.37	83.133	<0.001
BG-trap	30	6.21	3.14	0.57	5.03	7.38		
Chengwen Jingling Trap at the same time as light trap	30	0.78	0.88	0.16	0.45	1.11		
Chengwen Jingling trap at the same time as BG-trap	30	0.14	0.20	0.37	0.06	0.21		
Total	120	2.05	2.95	0.27	1.52	2.58		

M, mean; SD, standard deviation; SE, standard error; CI, confidence interval; LL, lower limit; UL, upper limit.

**Table 7:** Pairwise comparison of mosquito trapping efficacy among surveillance under concurrent operation periods.

Methods(I)	Methods(J)	M(I-J)	SE	*P*	95%CI	
					LL	UL
Light trap	Chengwen Jingling trap at the same time as light trap	0.30	0.21	0.645	−0.28	0.89
BG-trap	Chengwen Jingling trap at the same time as BG-trap	6.07^*^	0.58	<0.001	4.45	7.68

^*^The significance level for mean differences was set at 0.05.

### 3.4 Seasonal distribution of mosquito density

**Figure 3:**
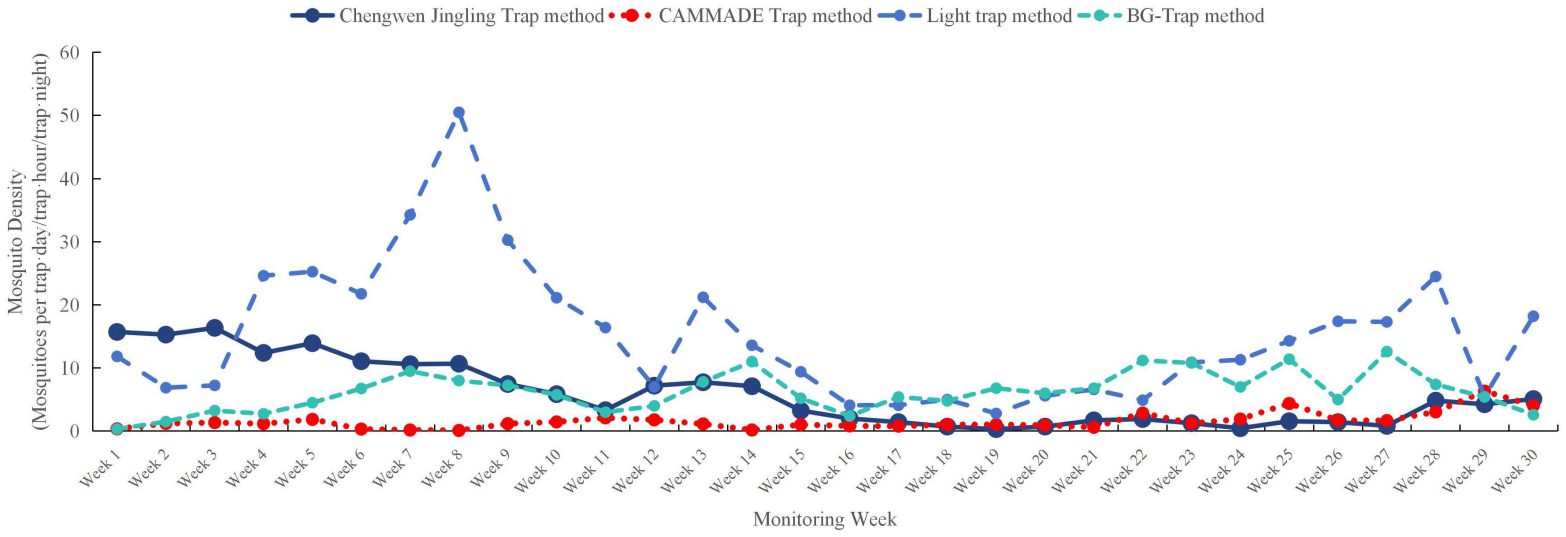
Seasonal dynamics of weekly mosquito density among surveillance methods in Zhejiang Province, 2025 (Note: mosquito density units: light trap method [mosquitoes/(trap-night)]; BG-trap method [mosquitoes/(trap-hour)]; Chengwen Jingling trap & CAMMADE trap method[mosquitoes/(trap-day)].

Weekly mosquito density (from Monday to Sunday) was used to analyze the seasonal distribution of the different methods. Week 1 corresponds to May 5–11, Week 30 corresponds to November 24–30, and so on. Temporal trend analysis revealed that the light trap method yielded higher mosquito densities during peak periods, but the timing of these peaks was also largely consistent with the BG-Trap method. The light trap method exhibited three density peaks in weeks 8, 13, and 28, whereas the BG-Trap method displayed relatively flat annual density profiles with minor peaks across multiple months. Chengwen Jingling trap surveillance initially recorded relatively high mosquito densities that decreased slightly over time with several intermittent minor peaks, followed by a modest increase around week 28. CAMMADE trap surveillance recorded lower mosquito densities than the other methods, but displayed several minor density peaks that were largely consistent with other surveillance methods (Figure 3).

The Dynamic Time Warping (DTW) algorithm was used to compare mosquito density variation trends between two intelligent monitoring devices (Chengwen Jingling and CAMMADE Trap) and the conventional surveillance methods. Both intelligent devices exhibited large numerical level differences (DTW normalized distance) compared to conventional methods (Table 8). Chengwen Jingling showed consistent trend patterns (directional correlation and sign consistency) with the light trap and BG-Trap methods, indicating reliable response to mosquito density fluctuations. Conversely, CAMMADE displayed opposite trend patterns when compared to the light trap method, suggesting poor responsiveness to mosquito density changes.

**Table 8:** Dynamic Time Warping (DTW) algorithm comparison of mosquito density between intelligent surveillance methods and conventional surveillance methods.

Classification	Intelligent surveillance methods	Conventional surveillance methods	DTW normalized distance	Directional correlation	Sign consistency^*^
Mosquito density	Chengwen Jingling Trap VS	Light trap	33.88^*^	0.34 (moderate (+))	69.00%
		BG-Trap	22.03^*^	0.22 (slight (+))	62.10%
	CAMMADE Trap VS	Light trap	54.04^*^	−0.34 (moderate (–))	37.90%
		BG-Trap	9.67^*^	−0.03 (none)	41.40%
*Aedes* mosquito density	Chengwen Jingling Trap VS	BG-Trap	14.39^*^	0.08 (none)	55.20%
	CAMMADE Trap VS	BG-Trap	17.81^*^	−0.13 (slight (–))	34.50%

^*^large DTW normalized distance; ^*^Sign consistency is defined as the percentage of adjacent time points with identical change direction; for instance, 69.0% indicates that 69.0% of the time points changed in the same direction.

The temporal trend consistency of *Aedes* mosquito densities was also detected between two intelligent monitoring devices and the BG-Trap method. Both intelligent monitoring methods showed large numerical-level differences (as measured by DTW normalized distances) compared with the BG-Trap method. CAMMADE trap showed opposite trends when compared with the BG-Trap method. These findings suggest that CAMMADE trap exhibits an inconsistent response pattern.

### 3.5 Accuracy of counting and identification by intelligent mosquito traps

**Figure 4:**
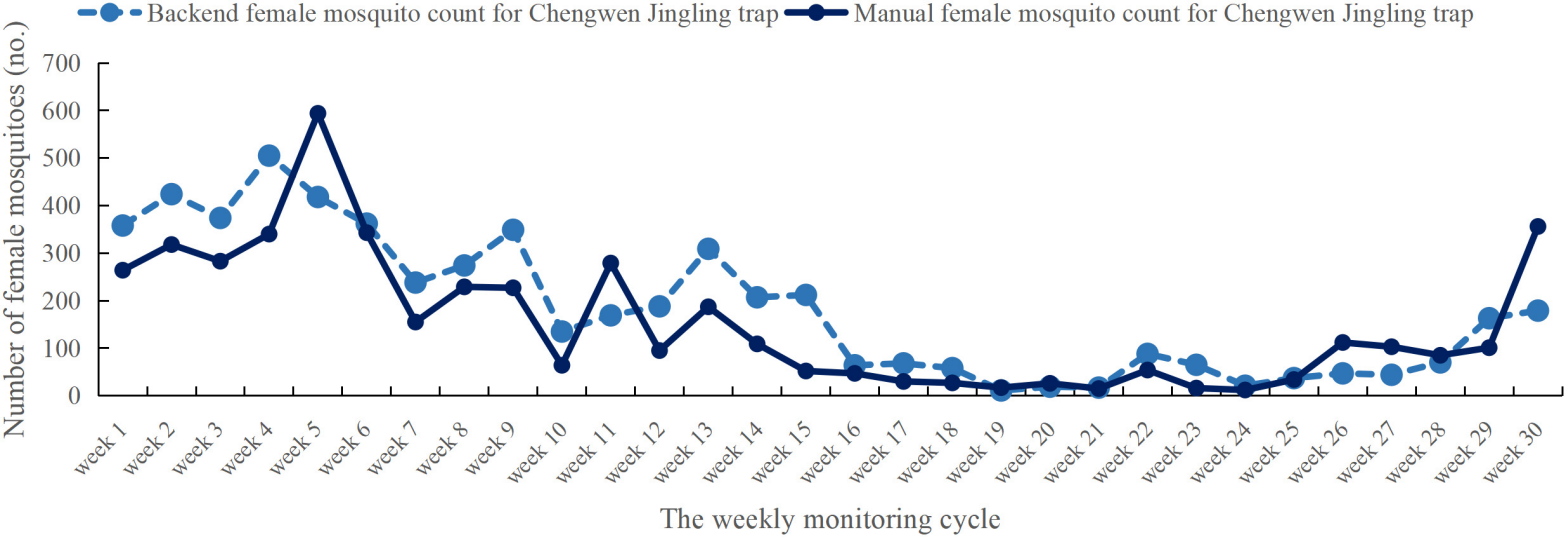
Temporal trend analysis of backend vs. manual female mosquito counts for Chengwen Jingling trap in Zhejiang Province, 2025.

**Figure 5:**
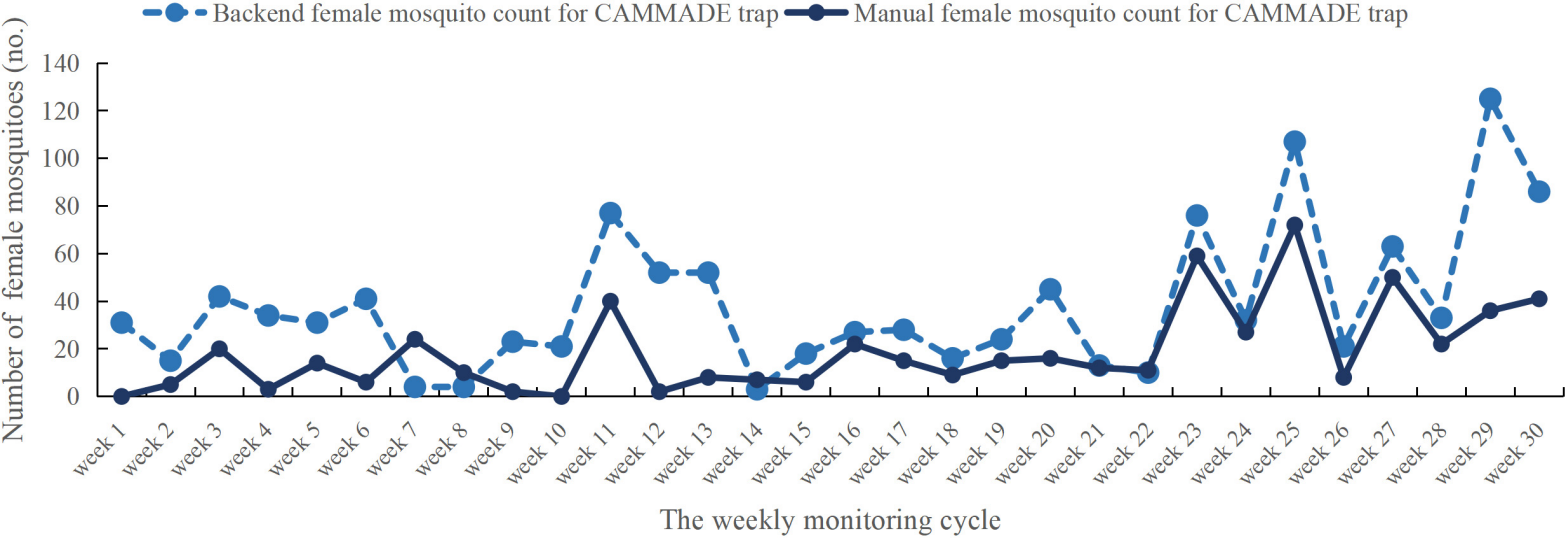
Temporal trend analysis of backend vs. manual female mosquito counts for CAMMADE trap in Zhejiang Province, 2025.

A manual correction protocol was applied to the backend data from intelligent monitoring systems. The weekly monitoring cycle was defined as the period from the previous mosquito collection from the intelligent monitoring devices to the current mosquito collection. During weekly routine surveillance, mosquitoes captured by the intelligent monitoring devices in the preceding monitoring cycle were retrieved and transported to the laboratory for manual counting and species identification. Seasonal distribution analysis was conducted on a weekly monitoring cycle basis between the number of female mosquitoes automatically counted by the backend of intelligent monitoring devices and the manually calibrated counts. The backend female mosquito count of Chengwen Jingling and CAMMADE trap showed a largely consistent trend with manual calibration counts (Figures 4, 5).

Both intelligent monitoring devices consistently overestimated mosquito densities relative to manual validation, though their error patterns differed markedly (Tables 9, 10). The relative error rate (RE) was calculated using the following formula: relative error rate = (measured value–true value) / True Value × 100%. The Chengwen Jingling trap substantially overestimated total mosquito abundance (RE: 73.88%), yet its female-specific counts and density estimates demonstrated considerably higher accuracy (RE: 25.67%), suggesting more reliable performance for the target surveillance metric. To address this overestimation, we propose an initial calibration factor of 1.26 (i.e., calibrated density = AI-reported density / 1.26). By contrast, while the CAMMADE trap achieved moderate accuracy for total captures (RE: 16.92%), its default assumption that all trapped mosquitoes are female resulted in severe overestimation of female density (RE: 105.34%).

**Table 9:** Backend counting results and manual identification results from intelligent monitoring devices in Zhejiang Province, 2025.

Methods	Data sources	Trap·day	No. of mosquitoes	No. of female mosquitoes	Mosquito Density	*Culex* species		*Aedes* species		*Anopheles* species		Others	
						female	male	female	male	female	male	female	male
Chengwen Jingling Trap	Automatic counts	971	11,730	5,473	5.64	4,858	5,305	615	915	0	37	0	0
	Manual counts	971	6,746	4,355	4.49	3,997	2,189	348	200	4	1	6	1
CAMMADE Trap	Automatic counts	778	1,154	1,154^*^	1.48	1,088	0	66	0	0	0	0	0
	Manual counts	778	987	562	0.72	536	402	26	23	0	0	0	0

^*^The CAMMADE trap backend defaults to assuming all captured mosquitoes are female.

**Table 10:** The relative error rate of automatic counts vs. manual counts for intelligent monitoring devices.

Methods	The relative error rate (%)					
	Total mosquitoes	Female mosquitoes	Total *Culex*	Female *Culex*	Total *Aedes*	Female *Aedes*
Chengwen Jingling trap	73.88	25.67	64.29	25.61	179.20	21.54
CAMMADE trap	16.92	105.34	15.99	105.56	34.69	102.99

Both intelligent devices were restricted to genus-level identification, with markedly divergent accuracy across taxa. *Culex* classification achieved moderate reliability, particularly for CAMMADE traps (RE: 15.99%) compared with Chengwen Jingling traps (RE: 64.29%), whereas *Aedes* identification incurred substantially higher error across both systems, with Chengwen Jingling traps showing particularly severe miscalculation (RE: 179.20 vs. 34.69% for CAMMADE). A contrasting pattern emerged for female-specific estimates: Chengwen Jingling traps demonstrated improved accuracy for female *Culex* (RE: 21.54%) relative to total captures, but remained problematic for female *Aedes* (RE: 76.72%); conversely, CAMMADE traps exhibited dramatically inflated sex-specific errors (female *Culex* RE: 102.99%; female *Aedes* RE: 153.85%) due to their default assumption that all captures were female.

## 4 Discussion

In our study, we conducted a pilot field project utilizing intelligent mosquito-monitoring devices to validate their trapping efficacy, automated counting, and species identification accuracy, while employing conventional surveillance methods at the same sites for comparative benchmarking. Our findings indicate that intelligent mosquito surveillance devices demonstrate considerable potential for enhancing automated mosquito identification and counting capabilities. Research has demonstrated that artificial intelligence has transformed the identification and surveillance of disease vectors through the automated recognition of mosquitoes, sand flies, ticks, and other medically significant arthropods (29, 30). A previous study reported that AlexNet accurately classified mosquito images from the genera *Aedes, Sabethe*s, and *Haemagogus*, achieving a classification accuracy exceeding 90% (31). Another investigation optimized CNN hyperparameters, achieving a classification accuracy of 97.3% in differentiating *Aedes* and *Culex* mosquitoes (32). A study introduced LarvaeCountAI, an open-source CNN-based tool designed for the automated counting of *Cx. annulirostris* larvae in laboratory videos, achieving an accuracy range of 96.25%−99.13% across ten test videos, with a mean accuracy of 97.88% (33). However, prior studies focused solely on taxonomy. However, in field studies, intelligent mosquito monitoring devices still suffer from duplicate counting and species misidentification errors. A field study indicated that the VECTRACK optical sensor, when paired with a standard BG-Mosquitaire suction trap, attained 99.8% accuracy in distinguishing target mosquitoes (*Aedes* and *Culex*) from non-target insects, 93.7% accuracy in classifying the two genera, and sex classification accuracies of 95% for *Cx. quinquefasciatus* and 92.1% for *Ae. aegypti* (34). In our study, the Chengwen Jingling trap achieved a RE of 25.67% for female mosquito counting and mosquito density, and 21.54% for *Culex* identification. In contrast, the RE for *Aedes* genus identification reached as high as 179.20%. The CAMMADE trap exhibited a RE of 16.92% for mosquito counting and density, with a striking RE of 102.99% for the identification of *Culex* genus female mosquitoes. Several factors may contribute to the above-mentioned errors. First, current devices exhibit limited attraction efficacy for *Aedes* mosquitoes, resulting in insufficient specimen captures. The scarcity of specimens then contributes to substantial errors in both automated counting and species differentiation. Second, in the field, non-target interference and duplicate counting of flying mosquitoes pose additional challenges that may compromise accuracy. Third, insufficient image resolution obscures key morphological traits, variable field lighting causes feature distortion, and morphological overlap between *Aedes* and other genera further complicates accurate identification. Fourth, the PSA modules in the AI head fail to mitigate these errors due to their reliance on global feature aggregation and the absence of an uncertainty rejection mechanism, which forces low-confidence predictions into a genus label and perpetuates misidentifications. A more critical limitation of the CAMMADE trap is its default assumption that all captured mosquitoes are female, which resulted in a systematic overestimation of density. This assumption is entomologically unjustified, as male mosquitoes are frequently present in field catches. In a real-world outbreak scenario, such overestimation could lead to premature activation of costly vector control measures, misallocation of limited public health resources, and false signals of escalating transmission risk. Consequently, this design flaw imposes significant limitations on the device's practical utility for ecological risk assessment. Addressing this hardware/software issue is therefore essential before the CAMMADE trap can be reliably deployed in outbreak surveillance. Seasonal distribution analysis revealed that the backend female mosquito counts from the Chengwen Jingling and CAMMADE traps exhibited a trend largely consistent with manual calibration counts. This consistency underscores the reliable stability of backend readings obtained from intelligent surveillance systems. Regarding the seasonal distribution of mosquito density among different methods, the Chengwen Jingling trap exhibited consistent trend patterns with the BG-trap methods, indicating a reliable response to fluctuations in mosquito density. While intelligent monitoring devices show promise for field monitoring applications, they do possess certain limitations. Future efforts should focus on leveraging AI capabilities, particularly deep learning algorithms, to enhance efficient detection, accurate identification, and continuous monitoring of target mosquito populations, all while minimizing resource expenditure (35).

The distinct ecological niches and behavioral traits of different mosquito species preclude the use of any single standardized traditional surveillance method for comprehensive population assessment. *Ae. albopictus* is diurnally active and highly responsive to CO_2_ and host-associated kairomones, whereas *Culex* species are predominantly nocturnal and strongly phototactic (36). Consequently, current surveillance protocols employ an integrated, multi-method approach that combines light traps with double-net or BG-trap methods to ensure effective monitoring of regional mosquito populations across diverse species and activity periods. In contrast, intelligent monitoring devices overcome these limitations by utilizing combined attractants (e.g., lures, CO_2_, and light) to achieve continuous day–night trapping. Therefore, the primary objective of comparing mosquito densities across different surveillance methods was to investigate whether standardized relationships exist between intelligent surveillance methods and conventional monitoring systems currently in operation, and to explore whether these comparative analyses could inform the development of control thresholds for intelligent monitoring devices. This comparison does not aim to accurately represent the trapping efficacy of each device, but rather to establish empirical conversions under operational conditions. In contrast, comparisons of trapping efficacy reflect each device's inherent trapping orientation and operational characteristics. Thus, both mosquito density comparisons and trapping efficacy evaluations are grounded in the context of currently operational mosquito monitoring systems, rendering the results potentially more applicable to real-world vector control programs.

The light trap method is currently the most widely employed technique for monitoring mosquito density in China, primarily due to its straightforward operation and its effectiveness in attracting *Culex* species (37). In our study, 97.14% of the mosquitoes captured using the light trap method were identified as belonging to the *Culex* genus, while only 2.69% were *Aedes* species. Consistent with our findings, previous field trials have reported that light traps captured significantly more *Culex* species than BG-Sentinel traps, whereas BG-Sentinel traps collected more *Aedes* species than light traps (36). Given the poor trapping efficiency of light traps for *Aedes* mosquitoes, this method is typically excluded from vector control assessment protocols for DF and CHIKF. Nevertheless, as *Culex* mosquitoes are major vectors of JE (38), the superior attraction of light traps to *Culex* species offers an important basis for informing JE prevention and control strategies. In our comparative analysis of weekly mosquito densities, the light trap method produced higher density estimates than other methods. Therefore, it remains a viable and widely utilized technique for assessing mosquito density, particularly for *Culex* species. The double net trap and BG-trap have demonstrated high attraction efficacy for *Aedes* mosquitoes and are widely used for monitoring *Aedes* density (39, 40). In current DF outbreak responses, a density threshold of ≤ 0.9 mosquitoes/(trap-hour) has been established as the control standard using the double net trap method (41). Previous studies have shown that BG-traps offer higher sampling efficiency, require less labor, and are free from human-bait attraction bias compared to double net traps. Consequently, BG-traps represent a more appropriate choice than double net traps for evaluating *Aedes* density (16). In our study, the BG-trap captured 75.19% of *Aedes* specimens, indicating strong attraction efficiency for this genus, and demonstrated superior trapping efficacy relative to the other methods evaluated. Therefore, consistent with the findings of a previous study (42), the BG-Trap method has proven to be one of the most effective tools for establishing mosquito density control thresholds in the prevention and control of DF and CHIKF epidemics. However, these methods above still have many limitations and are labor-intensive, which imposes substantial logistical burdens and workloads on grassroots staff.

When full-day operational data from the intelligent monitoring devices were adopted, the intelligent monitoring devices showed the lowest overall trapping efficacy among all methods evaluated. However, when comparing mosquito trapping efficacy during the same time period, no statistically significant difference was observed between the Chengwen Jingling trap and the light trap. This indicated that the low mosquito density during certain time periods lowered the full-day trapping efficacy of the intelligent monitoring devices. Relative to the light trap method, the Chengwen Jingling trap was enhanced with an attractant for *Ae. albopictus*, which increased the capture of *Aedes* mosquitoes. No statistically significant differences in weekly mosquito densities were observed among the BG-trap and Chengwen Jingling trap methods. Nevertheless, the Chengwen Jingling trap underperformed compared to the BG-trap method during the same time period, suggesting that their attractiveness to *Aedes* species requires further optimization. All of these findings suggested a potential framework for developing control thresholds tailored to intelligent monitoring devices. However, given the current error rates in automated identification and counting, direct application of AI-generated data for threshold-based decision-making remains premature. Therefore, before these devices can be used for autonomous threshold triggering, a minimum precision threshold for mosquito identification and counting (e.g., >90%) should be required, and this threshold must be validated through field experiments and adjusted according to real-world conditions. To address this limitation, we propose applying a calibration factor to align AI-generated data with manual standards. Specifically, as the Chengwen Jingling trap exhibited a RE of 25.67% for density estimation, we propose an initial calibration factor of 1.26 (i.e., calibrated density = AI-reported density / 1.26). This calibration factor should be refined through multi-season, site-specific concurrent comparisons between AI and manual methods. Furthermore, until the above accuracy benchmarks are met, control thresholds for intelligent monitoring devices must remain provisional and subject to manual verification.

The present study is characterized by several notable strengths. We are among the pioneers in conducting field trials of intelligent mosquito surveillance devices. The simultaneous implementation of conventional density monitoring at the same field sites enabled direct comparative assessments of density measurements and trapping efficiencies across different methodologies. Additionally, we conducted manual validation of the automated counting and taxonomic identification outputs from the intelligent surveillance system, quantifying the RE associated with these monitoring instruments. Despite these strengths, our study still has several limitations that must be acknowledged. The spatial coverage of our selected surveillance sites was restricted to five locations, which is insufficient for establishing conclusive control threshold ranges that correlate between intelligent mosquito surveillance devices and conventional monitoring approaches. The limited number of sites constrains the generalizability of our findings, and future research should expand the spatial scale to validate these preliminary results. Additionally, the current intelligent monitoring devices exhibit low attraction efficacy for *Aedes* mosquitoes, resulting in limited capture numbers. This scarcity of specimens contributes to substantial errors in both automated counting and identification processes. Furthermore, the identification capability is restricted to the genus level and cannot achieve precise species-level classification; some instruments even lack sex differentiation functionality. It should also be noted that the various surveillance methods operate under different trapping durations and periods and may capture distinct mosquito species compositions, potentially confounding cross-method comparisons. To address these technical constraints, future iterations of the monitoring system should prioritize enhancing attractant formulations and refining identification algorithms. Additionally, further research should focus on comprehensively evaluating the performance of intelligent monitoring instruments, specifically in terms of establishing standardized thresholds for mosquito surveillance and developing comparative metrics for trapping efficiency.

In conclusion, AI-based mosquito monitoring technologies demonstrate significant potential for the modernization of vector surveillance programs. Their ability to automate mosquito identification and counting, along with real-time data transmission, greatly enhances the intelligence of mosquito monitoring. However, current technical limitations must be acknowledged more explicitly. Challenges remain in improving trapping efficacy, particularly for *Aedes* species, as well as enhancing algorithm accuracy for automated counting and identification under field conditions. Therefore, the transition from manual to AI-driven monitoring should be framed as a hybrid approach in the near future, rather than a full replacement. During this transitional period, integrating AI as a supportive tool alongside conventional methods will be essential to realize its benefits while mitigating the risks associated with premature sole reliance on automated systems.

## Data Availability

The original contributions presented in the study are included in the article/supplementary material, further inquiries can be directed to the corresponding author.
